# Prospects and challenges regarding biosurfactants in advancing the petroleum industry

**DOI:** 10.3389/fbioe.2025.1697361

**Published:** 2026-01-08

**Authors:** Ojasvini Ahluwalia, Niketan Patel, Nikhil Narmeta, Sandra Guzman Sanchez, Alexandre Soares Rosado

**Affiliations:** 1 Department of Bioengineering, McGill University, Montreal, QC, Canada; 2 Biological and Environmental Science and Engineering Division, King Abdullah University of Science and Technology, Makkah, Saudi Arabia; 3 Department of Biotechnology, University Institute of Engineering and Technology, Panjab University, Chandigarh, India

**Keywords:** biosurfactants, oil industry, environmental sustainability, microbial-enhanced oil recovery, biometallization, biodesulfurization

## Abstract

As the global population increases, the petroleum industry is experiencing exponential increase in energy demands. However, the petroleum industry faces a myriad of problems concerning production, operation, sustainability, climate change and broader environmental implications. Traditionally, industry has relied on synthetic surfactants to address these challenges. However, owing to their toxicity, nonbiodegradability, and potential ecological leaching, the industry is increasingly shifting toward natural surfactants. Biosurfactants have numerous applications in the petroleum industry as enhanced oil recovery agents, emulsification and demulsification agents, anticorrosive agents, and biocides for sulfate-reducing bacteria. Biosurfactants are proven to be versatile, stable, and valuable biochemical tools capable of modernizing and mitigating challenges in the petroleum industry while promoting sustainability. To realize their potential, future applications and commercialization of biosurfactants require specific research and technological advancement that can increase their production. This review explores the challenges faced by the petroleum industry and the classification, properties, applications, market share, patents, and promising prospects of biosurfactants in various petroleum industry sectors.

## 1 Introduction

Petroleum is a critical raw material for energy production, and heating (Kashif et al., 2022; Othmani et al., 2022). Global petroleum production was projected to grow by 102.5 million barrels/day (b/d) in 2023, 103.2 million b/d in 2024, and 105.9 million b/d by 2025, whereas liquid fuel consumption was 101.9 million b/d, 102.9 million b/d and 105.1 million b/d, respectively, in those years (Renewables became the second-most prevalent U.S. electricity source, 2020). This imbalance will gradually deplete oil inventories, with current reserves estimated to last 4 decades (Kashif et al., 2021).

The petroleum industry faces major challenges, including limited reservoirs, suboptimal extraction of light crude, corrosion, losses during processing, difficulty in utilizing heavy crude, and complexities in transportation, storage, and oil-spill management (Al Madan et al., 2025; Ray et al., 2025). The Russia–Ukraine conflict introduced major volatility to the global energy market. During the initial weeks of the conflict, oil prices rose by 40%, remaining 27% above prewar levels a year later (Adolfsen et al., 2022). During the initial weeks of the Russia–Ukraine conflict, prices surged substantially, with oil prices rose by 40%, remaining 27% higher than the prewar price, coal by 130%, and gas by 180% (Adolfsen et al., 2022).

Novel techniques have emerged to address the increasing petroleum demands and mitigate industrial challenges. The use of surface-active agents (surfactants) in industrial processes, such as biodegradation, emulsification, antifoaming, waterproofing, cleaning agents, and enhanced oil recovery (EOR) techniques, is increasing exponentially, specifically due to their ability to act on the oil–water interface owing to their hydrophilic and hydrophobic moieties (Karthick et al., 2019; Myers, 2020; Manga et al., 2021; Narukulla and Sharma, 2024). The global surfactant market was valued at approximately $45.15 billion to $49.6 billion in 2024 and is expected to grow to between $68.05 billion and $72.14 billion by 2032, with a compound annual growth rate (CAGR) of around 4.9%–5.3% (Biosurfactants market size, share, trends, 2025).

However, synthetic surfactants are slowly polluting the environment due to their nonbiodegradable nature and high toxicity to biological entities, such as soil microbiomes, insects, aquatic species, and even humans (Manga et al., 2021; Ding et al., 2022; Li et al., 2025). According to research conducted in the South China Sea, anionic surfactants were found to comprise 57% and 43% of the surface microlayer and subsurface water, respectively (Uning et al., 2022). Moreover, Mousavi and Khodadoost (2019) reported decreased chlorophyll content in an aquatic fern plant (*Azolla pinnata)* exposed to sodium lauryl sulfate, a surfactant. Furthermore, anionic surfactants caused larval abnormalities and high cortisol levels in zebrafish (Rahimi et al., 2020).

Biosurfactants are a class of amphiphilic biomolecules produced on the cell surfaces of microorganisms that are observed at air−solid, liquid−solid, or polar−nonpolar media interfaces (Kourmentza et al., 2017; Markande et al., 2021; Hsu et al., 2025). Microorganisms produce these biosurfactants to improve their survival capabilities in extreme conditions by enhancing the availability of hydrophobic immiscible substrates, biofilm formation, and pathogenicity. These molecules have several attributes, including detergency, emulsification, foaming, and dispersion, and they are known to have the intrinsic property of reducing the interfacial tension (IFT). Compared with their chemical counterparts, biosurfactants have several advantages, such as bioavailability; biocompatibility; high selectivity; environmental sustainability; and tolerance to extreme temperature, pH, and salinity (Jahan et al., 2020; Mohanty et al., 2021; Hsu et al., 2025). Biosurfactants are extensively used in several industries, such as petrochemicals, pharmaceuticals, mining, beverages, cosmetics, nanotechnology, textiles, agriculture, and food processing (Drakontis and Amin, 2020; Ingsel et al., 2022; Hsu et al., 2025). In the petroleum industry, biosurfactants are multifunctional compounds used to efficiently eliminate oil sludge from storage tanks, facilitate microbial-enhanced oil recovery (MEOR), manage and remediate oil spills, and enable the transportation of heavy crude oil *via* pipelines (Drakontis and Amin, 2020; Nagtode et al., 2023; Hsu et al., 2025).

Although biosurfactants offer clear environmental advantages over conventional surfactants, there is a comparative trade-off in operational and economic parameters. Production of biosurfactants often utilizes synthetic or semi-synthetic procedures that may involve hazardous solvents, toxic acid or base catalysts, and energy-intensive downstream processing steps, leading to environmental concerns and low process efficiency (Thakur et al., 2024; Sharma and Lamsal, 2025; Hsu et al., 2025). Even though recent studies have explored the use of enzyme-assisted synthesis and green purification methods, the primary disadvantages remain their high production costs and slower reaction rates compared to their chemical counterparts. Biosurfactant production costs typically range from USD 5 to 20 per kg, whereas conventional surfactants can be produced for around USD 2 per kg (Sharma and Lamsal, 2025). Moreover, biosurfactant yields are often limited by substrate availability, fermentation conditions, and purification complexity. Hence, despite their superior biodegradability, lower toxicity, and sustainability profile, the adoption of biosurfactants in large-scale industries remains restricted, as customers are generally unwilling to pay a premium for comparable performance (Hsu et al., 2025; Sharma and Lamsal, 2025). Continued efforts in strain improvement, utilization of low-cost agro-industrial wastes, and process intensification are therefore crucial to bridge this economic gap and enhance the feasibility of biosurfactant commercialization (Hsu et al., 2025).

This review aimed to discuss the significance and categorization of biosurfactants, their distinctive properties relevant to the oil and petroleum industry, and their applications within the oil and petroleum sector.

## 2 Biosurfactants

Due to the disadvantages of synthetic surfactants and advancements in sustainable technology, at present, the focus is on natural and biodegradable compounds, such as biosurfactants. Biosurfactants are primarily synthesized by aerobic microorganisms in aqueous media with carbohydrates, hydrocarbons, or fats as their carbon source (Carolin et al., 2021; Farias et al., 2021; Eras-Muñoz et al., 2022; Hsu et al., 2025). Their unique structure allows the biosurfactant to modulate the IFT. Biosurfactant production is a complex process that serves various ecological and physiological purposes, helping microbial communities to adapt to and survive in various environmental sectors (Mohanty et al., 2021; Eras-Muñoz et al., 2022; Ingsel et al., 2022; Hsu et al., 2025). Biosurfactants are predominantly synthesized by microorganisms belonging to the *Pseudomonas*, *Bacillus*, *Candida*, *Rhodococcus*, *Marinomonas*, *Burkholderia*, *Sphingomonas*, *Cobetia*, *Acinetobacter*, and *Lactobacillus* genera (Phetcharat et al., 2019; Schultz and Rosado, 2019; Nikolova and Gutierrez, 2021; Hsu et al., 2025).

### 2.1 Factors influencing biosurfactant production

The yield of biosurfactant production is greatly influenced by the availability of nutrients and metabolic cues, especially the carbon and nitrogen sources and molar ratio C/N. Carbon sources (e.g., hydrocarbons, molasses, and waste oils) improve yield mainly by acting as immediate metabolic precursors of the biosurfactant’s hydrophobic tail (Solomon and Vishnu, 2025; Sharma and Lamsal, 2025). In most instances, these complex organic substrates, particularly lipids or long-chain alkanes, induce microbial processes specifically aimed at their utilization and, in the process, also result in the overproduction of the biosurfactant as a solubilizing agent as well as a detoxification process. On the other hand, nitrogen sources (ammonium compounds and peptones) facilitate microbial growth as well as the total cell concentration, indirectly enhancing the overall biosynthetic capacity. Optimal C/N ratios vary by bacterial strain and influence metabolite synthesis (Jahan et al., 2020; Mohanty et al., 2021). The C/N ratio usually behaves as a metabolic switch, such that a high C/N ratio (excess carbon, nitrogen limitation) most often redirects energy from cell growth to the production low-value agro-industrial waste streams (i.e., molasses, waste frying oils, and peptones from chicken feather or corn steep liquor) as the major C/N sources, producers can attain high yields while at the same time solving waste valorization, significantly reducing raw material expenses, environmentally friendly production, and driving the industry towards a circular bioeconomy (Sharma and Lamsal, 2025). Metabolic regulators like particular inducers like vegetable oil boost production while inhibitors EDTA and antibiotics hinder microbial growth of secondary metabolites, such as biosurfactants. This optimization has strong industrial importance for minimizing production costs and supporting sustainability (Pathania and Jana, 2020). Table 1 presents examples of biosurfactants; their microbial sources; and the effect of various carbon sources, nitrogen sources, C/N ratios and metabolic regulators on their yield.

**TABLE 1:** Effects of various factors on biosurfactant yield.

Factor	Biosurfactant	Microbial source	Source	Yield (mg/L)	References
Carbon	Glycolipoprotein	*Lactobacillus* *plantarum* G88	Glycerol	232	Mouafo et al. (2018)
	Lipopeptide	*Bacillus subtilis* ICA56	Glycerol	1,290	De França et al. (2015)
	Rhamnolipid	*Enterobacter cloacae*	Glucose	261	Harikrishnan et al. (2022)
	Sophorolipid	*Starmerella bombicola*	Sunflower acid oil	515	Jadhav et al. (2019)
Nitrogen	Glycolipid	*Rhodotorula* sp. CC01	Landfill leachate	163.33	Lin et al. (2019)
	Lipopeptide	*Bacillus subtilis Natto*	Urea	211	Leow et al. (2023)
	Rhamnolipid	*Pseudomonas aeruginosa* ATCC 9027	Ammonium nitrate	612	Wu et al. (2019)
	ND^a^	*Bacillus velezensis* ASN1	Ammonium chloride	818	Nair et al. (2019)
C/N Ratio	Lipopeptide	*Bacillus nealsonii*	Glycerol/ammonium nitrate	1,300	Phulpoto et al. (2020)
	Rhamnolipid	*Achromobacter* sp. (PS1)	Beef extract/sodium nitrate	413	Joy et al. (2019)
	ND^a^	*Bacillus* sp. BMN 14	Glucose/ammonium nitrate	246	Heryani and Putra (2017)
Inducer	Sophorolipid	*Basidiomycetes* yeast of *Rhodotorula* sp. VITJzN03	2% olive oil	7,000	Salam and Das (2013)

^a^ Not determinate.

### 2.2 Characterization

**FIGURE 1:**
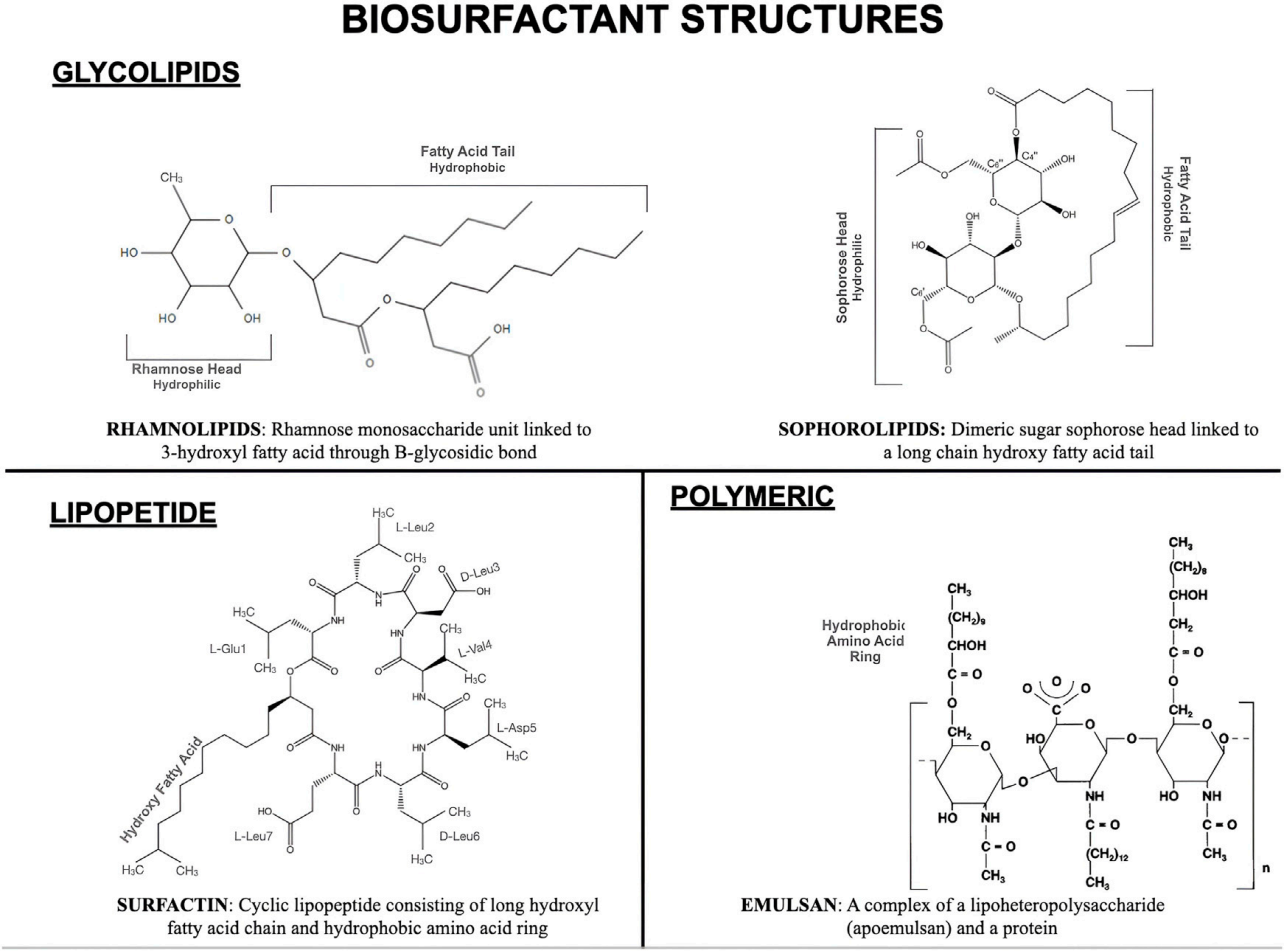
Structures of the key biosurfactants.

Biosurfactants can be anionic, cationic, or neutral in nature, depending on the presence of amine groups. The hydrophobic part of the biosurfactant typically contains a long-chain fatty acid, whereas the hydrophilic part can be a carbohydrate, cyclic peptide, amino acid, phosphate carboxyl, or alcohol (Farias et al., 2021; Nikolova and Gutierrez, 2021; Hsu et al., 2025). These surfactants can be categorized into two broad groups based on their molecular weights (low or high). Glycolipids, lipopeptides, and phospholipids are low-mass surfactants, whereas polymeric and particulate surfactants are high-mass surfactants (Silva et al., 2014; Sarubbo et al., 2022; Selva Filho et al., 2023). Figure 1 illustrates the structures of the essential biosurfactants.

### 2.3 Critical properties of biosurfactants in the petrochemical industry

Biosurfactants exhibit broad-spectrum properties owing to their unique and diverse structures.

#### 2.3.1 Critical micelle concentration (CMC)

**FIGURE 2:**
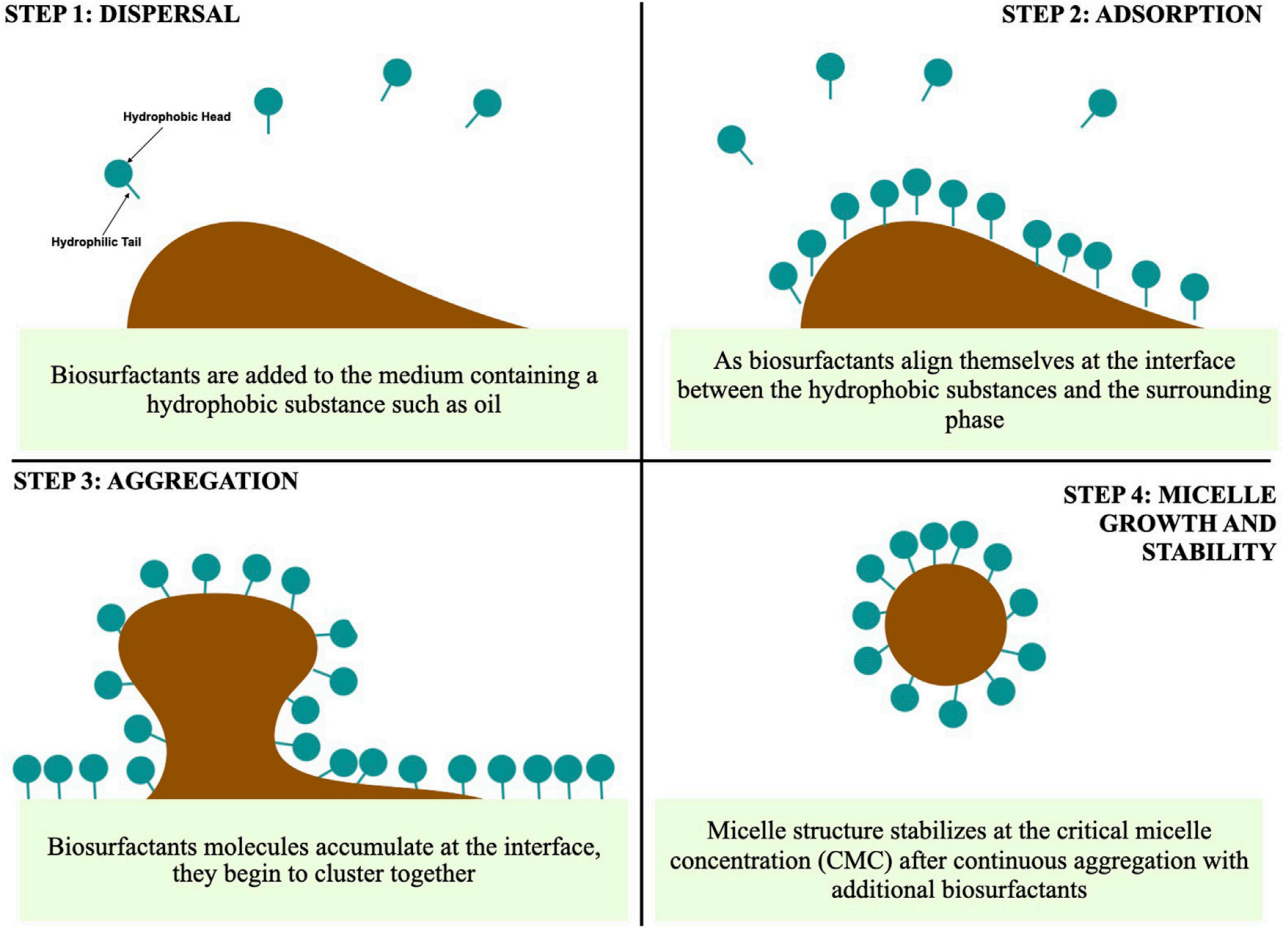
Micelle formation steps using biosurfactant (Image prepared using Biorender.com).

Minimum surfactant concentration required for effective surface and IFT reduction is the CMC, which directly affects the surface properties and performance. Biosurfactants typically have CMC values lower than those of chemical surfactants due to their complex molecular structures, enabling micelle formation at low concentrations (Chaprão et al., 2015; Santos et al., 2016; Kashif et al., 2022; Hsu et al., 2025 Santosetal., 2016). Synthesized by microorganisms to adapt to various environmental conditions, biosurfactants exhibit efficient functionality at lower concentrations and are biodegradable, minimizing their environmental impact (Patowary et al., 2017; Hentati et al., 2019). Figure 2 shows the mechanism of micelle formation at the CMC point by biosurfactants.

#### 2.3.2 Emulsification and demulsification

Emulsification involves creating stable mixtures of immiscible liquids, such as oil and water, facilitated by biosurfactants that reduce the IFT through their hydrophilic and hydrophobic interactions (Kashif et al., 2022; Kugaji et al., 2025). Low-molecular-weight biosurfactants, such as glycolipids and rhamnolipids, excel at emulsification and surface tension reduction, whereas high-molecular-weight biosurfactants are less effective in reducing surface tension but have high emulsification rates (Esmaeili et al., 2021; Al-Sakkaf and Onaizi, 2023). Biosurfactants also exhibit demulsification capabilities for treating waste emulsions, with applications in hydrocarbon degradation and remediation. Biosurfactants break oil-water emulsions by adsorbing at the interface, reducing surface tension, and displacing stabilizing agents such as asphaltenes or solids. This weakens electrostatic repulsion between droplets, promoting coalescence and phase separation, resulting in efficient demulsification. Chemical surfactants, however, often act through harsh solvents or surfactant exchange mechanisms and can leave toxic residues (Kugaji et al., 2025; Hu et al., 2025). Moreover, biosurfactants created by *Pseudomonas aeruginosa* and *Candida sphaerica* efficiently demulsify motor oil and reduce surface tension (Luna et al., 2015; Gidudu and Chirwa, 2020). For instance, field trials conducted by Hu et al. (2025) revealed that di-rhamnolipid fermentate achieved 99% demulsification at 50 °C lower than conventional polyether agents. Thereby, reducing energy costs by 15.14% and annual expenses by 31.31%, along with 150,000 tons of wastewater recovery.

#### 2.3.3 Tolerance to extreme temperature and pH

Biosurfactants tolerate extreme temperatures and pH due to their stable molecular structures, such as peptide and fatty acid chains, and microbial thermal stability from enzymes and hydrophobic regions (Mehetre et al., 2019; Kashif et al., 2022; Li et al., 2025). Biosurfactants gain their pH tolerance from charged functional groups, robust bonds, and structural adaptability to environmental changes, maintaining activity under harsh conditions (Derguine-Mecheri et al., 2018; Teixeira Souza et al., 2018; Ram et al., 2019). These properties make biosurfactants valuable in the oil and petroleum industries for enhancing oil recovery under extreme conditions. Moreover, biosurfactants maintain stability and activity from −20 °C to 200 °C and from pH 2 to 12 (de Almeida et al., 2016; Li et al., 2025).

#### 2.3.4 Biodegradability

Biosurfactants are natural and biodegradable. Their molecular structures, including lipids, peptides, or carbohydrates, are metabolizable by microbes with specialized enzymes (Jimoh and Lin, 2018; Kashif et al., 2022; Zhao et al., 2021). Emulsan polymerase, for example, inactivates emulsions by cleaving the polysaccharide backbones (Dos Santos et al., 2016). Furthermore, biosurfactants reduce the environmental impact on the petroleum industry by facilitating biodegradation of oil spills (Ali Khan et al., 2017; Selva Filho et al., 2023).

#### 2.3.5 Dispersion

In the petrochemical industry, biosurfactants function as natural dispersants by reducing the cohesive force between similar particles to inhibit aggregation. This dispersion property enables biosurfactants to desorb the hydrophobic organic crude oil compounds from rocky surfaces, resulting in an EOR procedure (Cai et al., 2016; Cai et al., 2021; Al-Sakkaf and Onaizi, 2023).

### 2.4 Applications of biosurfactants in the petroleum industry

Biosurfactants offer sustainable and efficient solutions to key challenges in the petroleum sector, including oil recovery from declining reservoirs, treatment of oily waste, and transport of viscous crude. Produced by diverse microorganisms, these surface-active compounds enhance microbial enhanced oil recovery (MEOR), aid in bioremediation of contaminated sites, break down oil sludge, and reduce the viscosity of heavy crude for easier pipeline transport (Gidudu and Chirwa, 2020; Malkapuram et al., 2021; Sarubbo et al., 2022; Zhao et al., 2025; Shaikhah et al., 2024). Some applications of biosurfactants in the petroleum industry are discussed below and summarized in Table 2.

**TABLE 2:** Biosurfactants and their applications in the petroleum industry.

Key activity	Biosurfactant	Microbial source	Application	References
Extraction	ND^a^	*Pseudoxanthomonas taiwanensis*	Additional 36.04% oil recovery in sand pack and 12.92% in core flooding experiments	Purwasena et al. (2024)
	Rhamnolipid	*Pseudomonas aeruginosa*	93.1% oil recovery at high temperature	Udoh and Vinogradov (2021)
	Surfactin	*Pseudomonas* sp.	15.4% increase in oil recovery	Panjiar et al. (2020)
	ND^a^	*Bacillus licheniformis*	13% residual oil recovery *via* interfacial tension reduction	Al-Sulaimani et al. (2010)
	Rhamnolipid	*Pseudomonas aeruginosa*	12% MEOR achieved for light crude oil	Câmara et al. (2019)
	Glycolipid	*Pseudomonas aeruginosa* HAK01	43% oil recovery	Khademolhosseini et al. (2019)
Corrosion	Glycolipid	*Bacillus subtilis* A1, *Streptomyces parvus* B7, *Pseudomonas stutzeri* NA3, *and Acinetobacter baumannii* MN3	87% inhibition efficiency	Rao and Mulky (2023)
	Glycolipid	*Pseudomonas fluorescens*	Significant delay in the corrosion of AISI 304 stainless steel	Dagbert et al. (2006)
	Glycolipid	*Pseudomonas sp*	87% in the corrosion rates	Parthipan et al. (2018)
	Rhamnolipid	*Pseudomonas cepacia*	Inhibited metallic corrosion and microbial biofilm	Da Silvaet al. (2024)
	ND^a^	*Bacillus* sp.	Reduced the corrosion rate in carbon steel ST 37 by 47%	Purwasena et al. (2019)
Pipeline transport of crude oil	Emulsan	*Acinetobacter calcoaceticus* PTCC1318	Tube cleaning with 100% crude oil removal	Amani and Kariminezhad (2016)
	Lipopeptide	*Kocuria rosea ABR6*	Reduced the crude oil transfer speed in the pipelines from 64 to 35 s	Akbari et al. (2021)
	Lipopeptide	*Bacillus amyloliquefaciens 6–2 c*	95.17% paraffin removal in the pipelines	Zhang et al. (2018)
	Surfactin	*Halomonas xianhensis* B2	Reduced pour point of crude oil and improved flow properties	El-Sheshtawy and Khidr (2016)
Bioremediation	Exopolysaccharide	*Acinetobacter calcoaceticus*	Enhanced hydrocarbon-degrading bacteria	Cappello et al. (2014)
	Lipopeptide	*Bacillus subtilis* CN2	82% degradation of motor oil PAH after 18 days of incubation	Bezza and Chirwa (2015)
	Rhamnolipid	*Pseudomonas cepacia* CCT6659	Increased biodegradation of oil by 70%	Soares da Silva et al. (2021)
	Surfactin	*Bacillus subtilis strain* O9	Significant removal of crude oil from sandy loam oil within 300 days	Cubitto et al. (2004)
Biocidal activity	Lipopeptide	*Bacillus* sp. H_2_O^−1^	Cell-membrane alterations and intracellular leakage in the SRB	Korenblum et al. (2012)
	ND^a^	*Bacillus* sp.	Inhibition of the corrosion-causing SRB	Zuo and Wood (2004)
	ND^a^	*Pseudomonas stutzeri* F01	Antimicrobial activity against SRB	Parthipan et al. (2018)

^a^ Not determinate.

#### 2.4.1 Microbial-enhanced oil recovery (MEOR)

With an increasing population, the demand for oil production is also increasing. Oil recovery is influenced by key physical factors, including the mobility ratio (M) and the capillary number (Nc). The Nc represents the ratio of the viscous forces to the local capillary forces, defined mathematically as the product of the fluid viscosity and velocity divided by the IFT. It serves as a measure of displacement efficiency, whereby reducing the IFT and increasing the viscous forces enhances the oil mobilization and recovery efficiency (Manjunath, 2022; Shaikhah et al., 2024). The M is a dimensionless parameter that defines the ratio of the mobility of the displacing fluid to that of the displaced fluid. For M >1, the displacing fluid has a higher mobility than the displaced fluid, or the displaced fluid has a higher viscosity, which can lead to poor sweep efficiency and channeling. To ensure effective oil displacement, it is crucial to maintain M of <1, where the displacing fluid has lower mobility, preventing fingering and bypassing of the oil (Manjunath, 2022; Guo et al., 2022). If the viscosity of water is lower than that of heavy oil, water seeps into the reservoir rock instead of effectively displacing the oil, leaving significant residual oil behind and reducing the recovery efficiency (Guo et al., 2022).

Primary extraction recovers 10%–15% of the oil using natural and artificial lifts. Secondary recovery, including waterflooding and gas injection, supplements the primary methods, increasing recovery by 10%–30% but leaving >65% of the oil trapped due to capillary forces and IFT (Nasralla and Nasr-El-Din1 2014; Samba et al., 2021; Shaikhah et al., 2024). The efficiency of oil recovery (Ero) is determined by the product of the macroscopic (volumetric) displacement efficiency (Evo) and the microscopic displacement efficiency (Edo), which is expressed as follows:

Ero=Edo×Evo (1)

Equation 1 indicates that the overall recovery efficiency depends on the effectiveness of displacing oil at the pore scale (microscopic efficiency) and the extent to which the injected fluid sweeps the reservoir volume (macroscopic efficiency) (Beteta et al., 2023). The EOR techniques address these limitations by employing heat, surfactants, microbial processes, or gas injections to reduce IFT, alter reservoir wettability, and mobilize hydrocarbons (Nasralla and Nasr-El-Din, 2014; Patel et al., 2015; Samba et al., 2021; Shaikhah et al., 2024). However, chemical surfactants face challenges, such as high costs, nonbiodegradability, and environmental concerns.

As a cost-effective and ecofriendly alternative, MEOR leverages microbial activities and metabolites, such as biosurfactants, to improve recovery rates, and is advantageous over chemical techniques due to its nontoxic nature and the use of inexpensive raw substrates, such as waste materials (Bachari et al., 2019; Mohsenatabar Firozjaii and Saghafi, 2020; Haq, 2021; Chen et al., 2023). Biosurfactants exhibit substantial potential in facilitating EOR through four primary mechanisms such as reducing the IFT between oil and rock surfaces, improving the porous media wettability, and inducing crude oil emulsification (Santos et al., 2016; Vishnyakov et al., 2020; Andrade et al., 2024). The MEOR techniques involving biosurfactants use three prominent strategies: 1) injection of *ex situ*-produced biosurfactants into oil reservoirs; 2) *in situ* injection of specifically chosen biosurfactant-producing microorganisms into the reservoirs; and 3) application of suitable nutrients to stimulate biosurfactant production *in situ* (Liu et al., 2020; Vishnyakov et al., 2020; Andrade et al., 2024).

**FIGURE 3:**
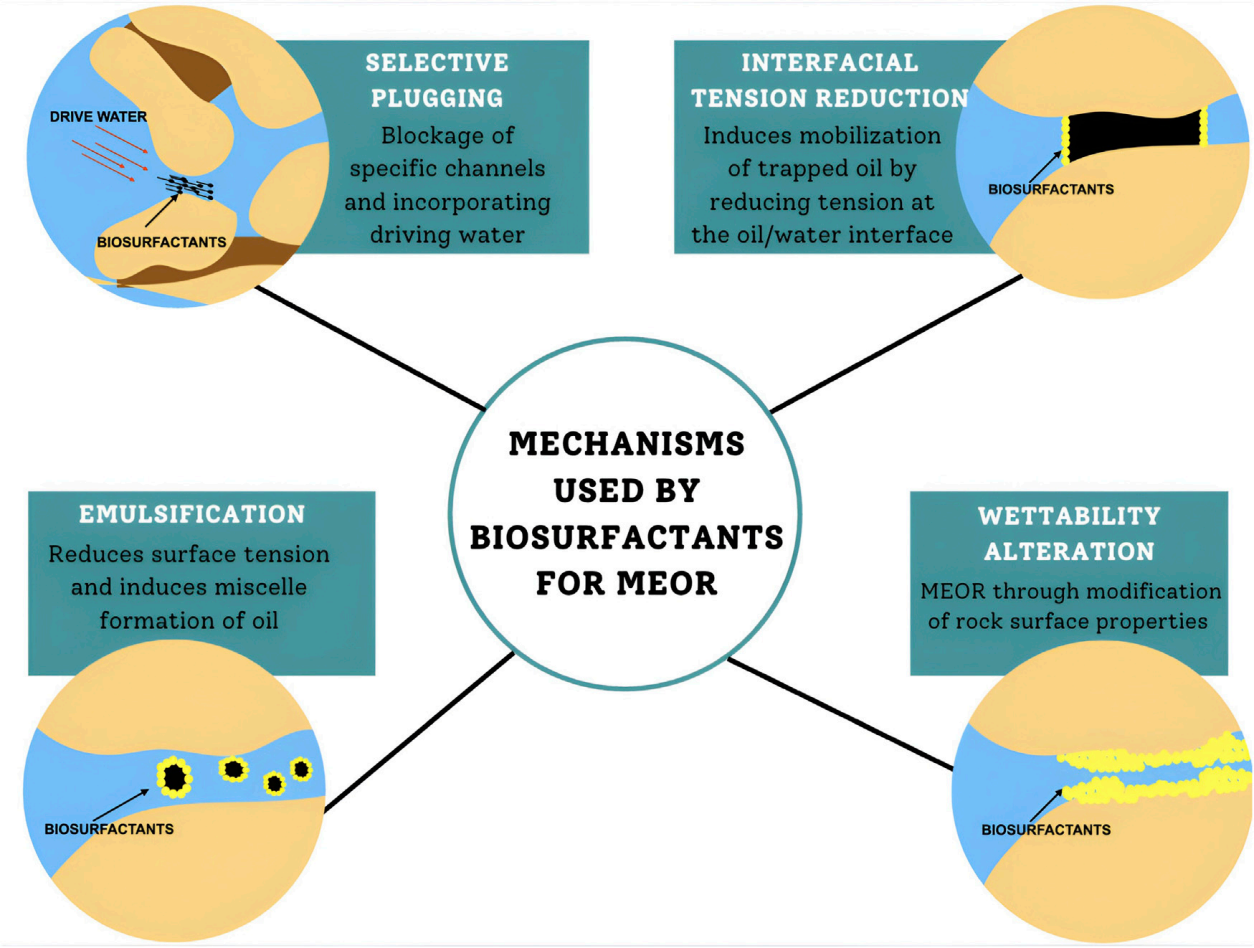
Biosurfactant mechanisms for microbial-enhanced oil recovery (MEOR).

A previous study demonstrated that when rhamnolipids are injected with controlled salinity brine, they achieved 93.10% oil recovery at 70 °C, whereas GreenZyme and brine reached 82.76% recovery at 23 °C (Udoh and Vinogradov, 2021). Similarly, Nazina et al. (2020) isolated the biosurfactant-producing microorganisms, *Rhodococcus metropolis* and *Gordonia amicalis* from Russian oil reserves, which enhanced MEOR by reducing surface tension and aiding hydrocarbon bioremediation. In a field trial in Saskatchewan, the injection of a nutrient solution boosted indigenous microbial activity and increased daily oil production by >200%, from 1.2 m^3^/days to 4.1 m^3^/days (Town et al., 2010). Figure 3 depicts the mechanisms used by biosurfactants for MEOR.

These findings collectively highlight the effectiveness of biosurfactant-mediated MEOR can enhance oil recovery by 10%–30% compared to the older methods. Rhamnolipids and lipopeptides are highly effective under high-salinity and temperature conditions. However, scalability and reservoir heterogeneity remain key challenges that must be addressed for field-wide applications.

#### 2.4.2 Anti-corrosion activity of the biosurfactants

Corrosion is a major issue in the petroleum industry, affecting material properties and costing >USD 60 billion annually worldwide (Groysman, 2017). Despite the protective coatings on petroleum storage tanks and pipelines, corrosion remains prevalent due to factors such as hydrogen sulfide (H_2_S) gas, water accumulation (Speight, 2014; Hassan-Beck et al., 2019). Refineries face additional challenges with desalting units and crude oil distillation units, where naphthenic acid and asphaltenes contribute to fouling and corrosion (Syafaat and Ismail, 2015; Ibrahim et al., 2018). Moreover, corrosion management strategies, including protective coatings and inhibitors, incur significant costs, making biosurfactants a promising alternative due to their ecofriendly properties (Zain Md et al., 2018; Fenibo et al., 2019).

Biosurfactants are ecofriendly alternatives to traditional surfactants, which are synthetic, petroleum-derived surface-active agents such as sodium lauryl sulfate and linear alkylbenzene sulfonates, due to their natural degradation and reduced toxicity (Akbari et al., 2018; Jahan et al., 2020; Mohanty et al., 2021). In petroleum, they prevent corrosion by reducing the surface tension, making it harder for corrosive agents to adhere to the metal. They also form protective films on metal surfaces, chelate metal ions that catalyze corrosion, and exhibit antimicrobial and antibiofilm activities, reducing microbially-influenced corrosion (Kip et al., 2017; Loto, 2017; Pal and Lavanya, 2022; Rao and Mulky, 2023).

The anti-corrosion potential of a biosurfactant produced by *Bacillus* sp. was investigated and found to be effective for reducing the corrosion rate in carbon steel ST 37 from 5.18 × 10^−5^ to 2.7 × 10^−5^ mm per year by biofilm eradication and prevention of the attachment of *Pseudomonas* sp. 1 and *Pseudomonas sp*. 2 on the steel surface (Purwasena et al., 2019). Parthipan et al. (2018) reported that a biosurfactant inhibited corrosion by an impressive 87%, which highlights the potential of *Pseudomonas* spp. biosurfactants as a powerful agent to stop biofilm-induced corrosion. Further, amino acid–based surfactants such as *N*-dodecyl asparagine, sodium *N*-dodecyl tryptophan, and sodium *N*-dodecyl histidine have demonstrated significant copper corrosion inhibition, with efficiencies of 81%, 82%, and 88%, respectively (Fawzy et al., 2023). Similarly, the biosurfactant produced by the yeast *Starmerella bombicola* ATCC 222214 effectively protected metal surfaces against both atmospheric oxidation and seawater-induced corrosion (Selva Filho et al., 2025). In another study by Faccioli et al., 2025, it demonstrates that the biosurfactant synthesized by *Pseudomonas cepacia* CCT 6659 exhibited a corrosion inhibition efficiency of 58.02% on carbon steel.

Overall, studies demonstrate that biosurfactants derived from *Bacillus* and *Pseudomonas* species can reduce corrosion rates in steel by up to 80%–90% by forming protective films and disrupting microbial biofilms, highlighting their potential as sustainable corrosion inhibitors compared to chemical surfactants.

#### 2.4.3 Enhanced transport of crude oil through pipelines

The growing demand for petroleum and the decline in light and medium crude oil resources have led to the extraction of heavy crude oil and bitumen, which now account for >70% of the global oil reserves. Heavy oil has an American Petroleum Institute (API) gravity of up to 20, whereas extra heavy oil/bitumen has an API gravity <10 and viscosity of ≤10^6^ mPa-s, making extraction, production, and transportation challenging due to the presence of asphaltenes, paraffin precipitation, and high viscosity. Conventional pipelining is limited to viscosities <200 cP, and brine increases the corrosion risk (Amani and Kariminezhad, 2016; Fenibo et al., 2019; Uddin et al., 2025). Techniques, such as dilution, heated pipelines, and pour point depressants, have been used to address viscosity and transportation issues but face limitations, such as cost and inefficiency. Recently, biosurfactants have emerged as an alternative that reduces viscosity, aids emulsification, and improves transportability through pipelines, even during shutdowns (Fenibo et al., 2019; Manga et al., 2021; Uddin et al., 2025).

*Acinetobacter calcoaceticus* PTCC1318 produced an emulsan biosurfactant, which was highly effective for reducing the surface tension of water to 24 mN m^−1^ and the IFT of water to 3 mN m^−1^. Moreover, this emulsan biosurfactant succeeded in tube cleaning, achieving 98% crude oil removal at 25 °C (Amani and Kariminezhad, 2016). EL-Sheshtawy and Khidr (2016) demonstrated that the biosurfactant produced by the strain *Halomonas xianhensis* (B2) significantly reduced the pour point of crude oil and could be used as an additive to improve the flow properties.

Both studies highlighted the that biosurfactants can effectively reduce crude oil viscosity, improve flowability, and enhance cleaning efficiency in pipelines, achieving up to 98% oil removal in laboratory setups.

#### 2.4.4 Environmental remediation using biosurfactants

The petroleum industry generates approximately 260,000 metric tons of waste annually, including the produced water, sludge, and contaminated equipment (de Almeida et al., 2016; Selva Filho et al., 2023; Raheja et al., 2025). Environmental contamination arises from oil spills, corroded tanks and pipelines, and industrial byproducts such as pharmaceuticals, plastics, and cosmetics. Pollutants such as waxes, asphaltenes, hydrocarbons, and heavy metals persist in the environment, exerting cytogenetic, mutagenic, and carcinogenic effects (El Gheriany et al., 2020; Selva Filho et al., 2023).

Bioremediation using biosurfactants is a promising solution due to their cost-effectiveness, biodegradability, and ease of use, making them ideal for environmental cleanup. The following sections explore their applications in bioremediation.

##### 2.4.4.1 Bioremediation of Marine environments

Oil spills have devastating effects on marine ecosystems. The hydrophobic nature of crude oil prevents it from mixing with water, forming a surface layer that blocks sunlight and oxygen (Cherrier et al., 2013; Nikolova and Gutierrez, 2021). The Deepwater Horizon spill in 2010 released approximately 134 million gallons of oil, severely impacting marine life. Similarly, the 1991 Persian Gulf spill caused substantial mortality among marine species. Conventional cleanup methods, such as mechanical containment and chemical dispersants, have limitations. Conversely, biosurfactants facilitate oil displacement and enhance biodegradation rates (Nikolova and Gutierrez, 2021; da Silva Correa et al., 2022).

The biosurfactants produced by the strain *P. cepacia* CCT6659, which was isolated from industrial waste, dispersed 96% of the oil in seawater and increased oil biodegradation by 70%. This biosurfactant also posed low toxicity to *Allium cepa* crops and freshwater species, such as *Poecilia vivipara* and *Anomalocardia brasiliana*, indicating that the biosurfactant has bioremediation applications without threatening plant and aquatic species (da Silva Correa et al., 2022). Farooq et al. (2024) observed that in marine oil-spill simulations, the biosurfactant-based MELs-T dispersant reduced interfacial tension to 0.01 mN/m and had more than 90% of dispersion efficiencies which was comparable to those of Corexit 9500A, while maintaining biodegradability and low toxicity.

##### 2.4.4.2 Bioremediation of contaminated soil

Oil spills affect marine environments, but most occur on land, hampering local vegetation. Soil acts as a sink for hydrocarbons from storage leaks, pipeline failures, and transport accidents (Devianto et al., 2020; da Silva Correa et al., 2022; Raheja et al., 2025). Low-molecular-weight hydrocarbons leach into groundwater or evaporate, whereas heavier compounds bind to the soil and degrade through organic matter. Moreover, factors, such as terrain, climate, and soil properties, influence bioremediation rates (Selva Filho et al., 2023).

These contaminants can persist for years, causing plant stress, reduced photosynthesis, altered leaf structure, and osmotic stress (Arellano et al., 2015; da Silva et al., 2024; Raheja et al., 2025). Human and animal exposure can lead to severe health effects, including cancer, asthma, and liver damage (Kim et al., 2013; Fenibo et al., 2019).

**FIGURE 4:**
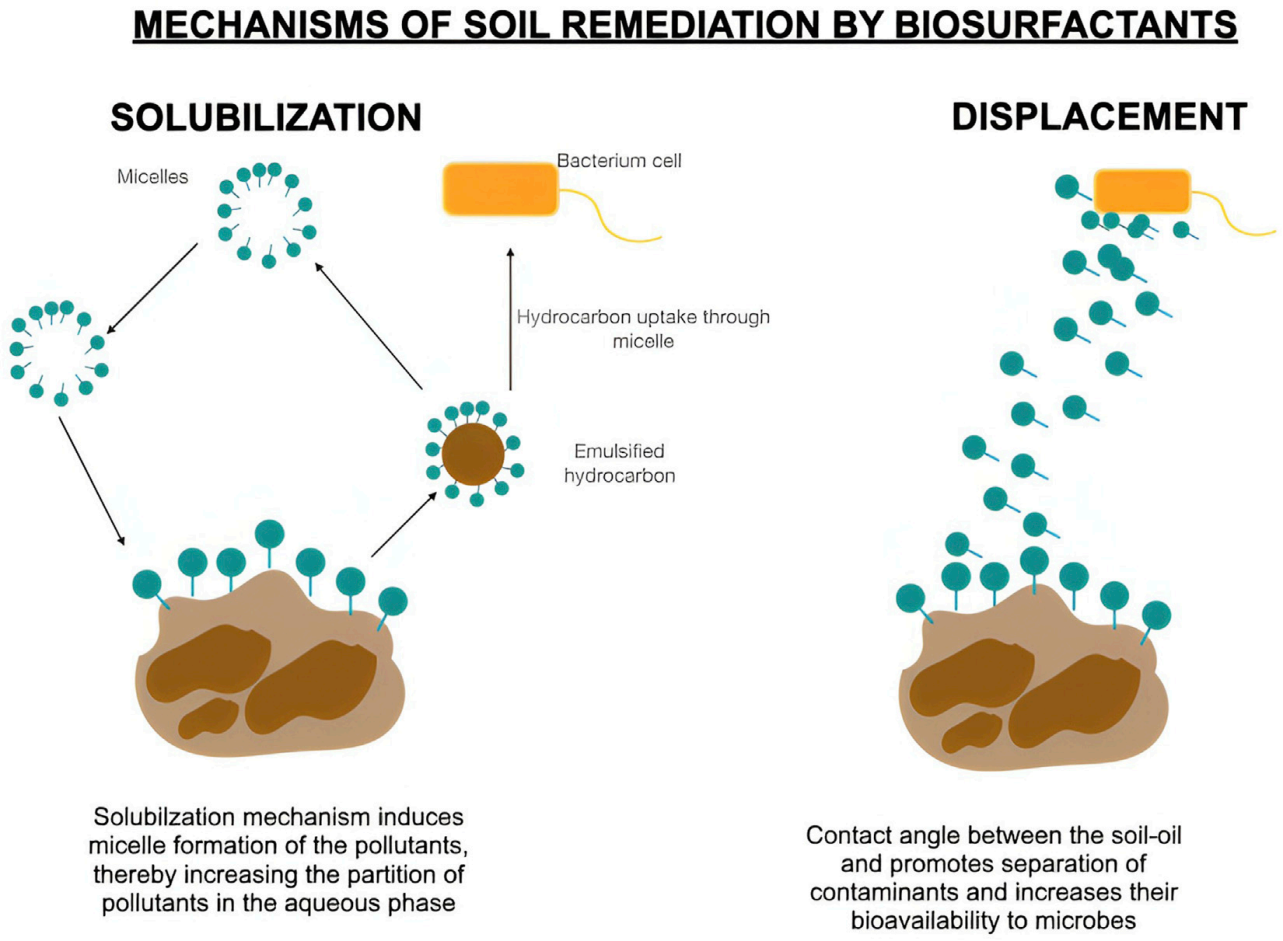
Mechanistic approaches of biosurfactant in soil remediation (Image prepared using Biorender.com).

Thus, bioremediation using biosurfactants is a promising soil remediation method. Biosurfactants enhance microbial degradation by solubilizing pollutants, forming micelles, and boosting enzyme activity (Fenibo et al., 2019; Sales da Silva et al., 2020; Raheja et al., 2025). For example, a *Bacillus* consortium reduced hydrocarbons by 43% in 15 days, whereas *P. cepacia* CCT6659 achieved 95% degradation in 35–60 days (Silva et al., 2014; Elenga-Wilson et al., 2021). As illustrated in Figure 4, biosurfactants facilitate soil remediation primarily through solubilization and displacement mechanisms.

Soil washing combines biosurfactants with water to mobilize or solubilize contaminants, enhancing pollutant removal through micelle formation and subsequent treatment (Sales da Silva et al., 2020). Biosurfactants from *Bacillus cereus* UCP1615 removed up to 87% of the petroleum products, whereas *Azotobacter vinelandii* emulsified and removed 48.89% of the hydrocarbons in 40 min (Durval et al., 2021; Devianto et al., 2020). In another study, a biosurfactantisolated from *Bacillus Sp* was able to remove more than 90% of the crude oil from contaminated soil within 48 h whereas only 70% of crude oil was removed with chemical surfactants, Tritonx-100 and CTAB. This study also highlighted that the mechanism of biosurfactant enhanced crude oil removal below CMC value (Suganthi and Bharathidasan, 2023). Biosurfactants offer an efficient and sustainable solution for remediating contaminated soil and water.

Studies have suggested that biosurfactant-assisted bioremediation improves hydrocarbon degradation by 40%–90% depending on microbial strain, substrate, and environmental conditions. The combined use of biosurfactants and microbial consortia significantly enhances pollutant solubilization and removal efficiency, making them highly promising for eco-friendly remediation strategies in marine and terrestrial environments.

#### 2.4.5 Biocidal activity of biosurfactants for sulfate-reducing bacteria

Sulfate-reducing bacteria (SRBs) are major contributors in oil-field souring and microbial-induced corrosion, producing H_2_S and sulfide ions during secondary oil recovery. Sulfide accelerates electrochemical and anaerobic corrosion, degrades crude oil quality, and increases operating costs by plugging wells, negatively affecting the MEOR processes. Sour corrosion, which is responsible for approximately 40% of petroleum industry corrosion, is especially severe at welded joints, leading to material cracking and leakage within hours (Zuo and Wood, 2004; Korenblum et al., 2012; Parthipan et al., 2018; Sivakumar et al., 2024). SRBs utilize hydrocarbons as electron donors, and the introduction of sulfate-rich water into reservoirs intensifies souring by promoting sulfide production. Corrosion typically begins with the adhesion of biofilms, which mature and degrade metal surfaces (Zuo and Wood, 2004; Parthipan et al., 2018). Biosurfactant injection counters this by enhancing sulfide-oxidizing bacterial activity, suppressing SRBs through competitive exclusion and biosurfactant production. Biosurfactants, specially lipopeptides, are known to disrupt the cell membranes and cell walls of SRB and retard the movement of ions inside and outside of the bacteria, leading to bacterial death. The lipid components of the biosurfactants are effective in dissolving the biofilms. Rhamnolipids inhibit the growth of biofilms and microbial cells during the doubling period of bacterial cell (Sivakumar et al., 2024). Biosurfactants also adsorb onto the metal surface, forming protective films, reducing oxygen/ion diffusion and thereby changing the redox or micro-environment so that it becomes toxic for SRB growth (Faccioli et al., 2025).

El-Sheshtawy et al. (2015) reported negligible growth of SRB in the culture media at 1% *Bacillus licheniformis* biosurfactant concentration. The biosurfactant also stimulated 16.6% oil recovery after its application. In another study, the treatment of SRBs with a lipopeptide biosurfactant synthesized by *Bacillus* sp. H_2_O^−1^ resulted in significant cell-membrane alterations and intracellular leakage (Korenblum et al., 2012). These findings emphasize the potential of biosurfactants lipopeptide and rhamnolipid biosurfactants as biocidal agents against SRBs, by disrupting cell membranes and inhibiting biofilm formation, thereby mitigating sour corrosion and improving oil recovery efficiency, particularly in the petroleum sector.

## 3 Market trends and commercialization challenges of biosurfactants

The global biosurfactant market was valued at USD 4.41 billion in 2023 and is expected to grow to approximately USD 6.71 billion by 2032, exhibiting an annual growth rate of 5.4%. Europe led this market in 2023, holding nearly half (48.53%) of the share, mainly due to stringent environmental regulations and a growing preference for sustainable and biodegradable surfactants (Aslam et al., 2024; Biosurfactants market size, share, trends, 2025). In the United States, the biosurfactant industry is also set to expand substantially, with its market expected to reach approximately USD 1.24 billion by 2032, driven by rising demand across the industrial, pharmaceutical, and oil recovery sectors. Since the COVID-19 pandemic, the market has seen an even greater shift toward ecofriendly alternatives, with increasing regulatory support and wider industrial applications driving this growth (Aslam et al., 2024).

Key companies involved in biosurfactant production include Jeneil Biotech, Soliance, Saraya, MG Intobio, Evonik, and AGAE Technologies, which are primarily based in North America, Asia–Pacific, and Europe. Evonik’s new facility in Slovakia is notably the world’s first dedicated plant for producing high-quality rhamnolipid biosurfactants, placing Evonik in a leading position due to its proprietary fermentation-based manufacturing processes (Evonik, 2024). Other companies, including AGAE Technologies, Jeneil Biotech, Biofuture, and TensioGreen, are actively scaling up rhamnolipid production for diverse applications, such as EOR, crude oil emulsification, and cleaning oil storage tanks (Wisjnuprapto et al., 2011; de Almeida et al., 2016; Aslam et al., 2024). Between 2017 and 2023, patent activity related to biosurfactants significantly increased, particularly within cosmetics, environmental applications, bioprocess innovations, and the petroleum industry, highlighting growing innovation and commercial interest (Nasser et al., 2024).

Despite its promising market growth, scaling biosurfactant from laboratory to commercial scale remains challenging. High production costs, largely due to expensive raw materials, inadequate bioprocess engineering, and limited microbial strain improvement, are significant barriers. For example, synthetic surfactants typically cost approximately 1–4 USD/kg, whereas biosurfactants, such as sophorolipids, cost approximately 34 USD/kg. Although such costs may be admissible in high-value, low-volume markets, such as pharmaceuticals or cosmetics, they pose a major hurdle in cost-sensitive industries, such as petroleum. Operational costs for biosurfactant production are primarily driven by raw materials (10%–80%), consumables (1%–50%), utilities (1%–30%), labor (5%–30%), facility maintenance (1%–40%), and miscellaneous expenses (<20%) (Domínguez Rivera et al., 2019; Babu et al., 2022; Muthaiyan Ahalliya et al., 2023). In addition, complex downstream purification and recovery processes further elevate overall production expenses. Other challenges include the limited understanding of biosurfactant toxicity toward environmental microbial communities, their reduced effectiveness under extreme environmental conditions, the potential for microbial-induced corrosion caused by SRB, and the evolving regulatory frameworks that can delay product approvals and raise compliance costs. Addressing these technical and economic challenges will be essential for biosurfactants to become viable, widely adopted alternatives to synthetic surfactants across diverse industries (Babu et al., 2022; Muthaiyan Ahalliya et al., 2023).

## 4 Advancements and optimization strategies for biosurfactant production

To address these challenges and optimize the implementation of biosurfactants in the petroleum industry, several innovative approaches can be explored, as discussed in this section.

### 4.1 Sustainable and renewable substrates

Production of biosurfactant depends on the quality and type of raw materials or substrates that the microorganisms will utilize as carbon and nitrogen sources. Conventional substrates, such as glucose, sucrose, vegetable oil, and glycerol, often have certain disadvantages, including high costs, limited availability, and unsustainable extraction and processing approaches. The utilization of conventional substrates contributes to almost 80% of the operational costs, whereas economic viability studies have shown a <50% return on investment (Domínguez Rivera et al., 2019; Babu et al., 2022). Furthermore, production of biosurfactants using such substrates adds to the release of greenhouse gases and pollution. Therefore, renewable and low-cost substrate alternatives are increasingly being evaluated for biosurfactant production. Since microorganisms are the primary producers of biosurfactants, the selected substrates must not only meet their nutritional requirements by maintaining an optimal balance of carbohydrates and lipids but also support proper microbial metabolism to ensure high-yield, quality biosurfactant production. This is where byproducts or waste products from the agriculture and dairy industry become valuable resources (Banat et al., 2010; Vieira et al., 2021; Lee et al., 2022; Shaikhah et al., 2024; Hsu et al., 2025).

Each year, approximately 1,300 million tons of agricultural waste is generated globally, contributing to approximately 3% of global greenhouse emissions, equivalent to nearly 1.2 gigatons of CO_2_ annually, with this figure steadily increasing (Alan and Köker, 2023). Rather than relying on incineration or landfilling for waste disposal, which exacerbates environmental concerns, this agricultural waste presents an opportunity to be repurposed for the sustainable and inexpensive production of biosurfactants (Domínguez Rivera et al., 2019; Alan and Köker, 2023). Sugars, molasses, plant oils, starch waste, whey, rice bran, fruit, vegetable waste, plant-oil extracts, distillery waste, olive oil mill effluents, wastewater, bagasse, oilseed cakes, straw, and stalks are some of the agroindustrial waste materials that have been evaluate as potential renewable biosurfactant substrates. These agricultural wastes are not only cost-effective but also excellent sources of carbohydrates, lipids, nitrogen, magnesium, manganese, phosphorous, iron, and other minerals, which increase the production and extraction of biosurfactants (Domínguez Rivera et al., 2019; Vieira et al., 2021; Lee et al., 2022; Shaikhah et al., 2024; Hsu et al., 2025). For example, molasses, a common byproduct of the sugarcane-processing industry, contains approximately 56% carbohydrates, 12% nonorganic matter, 2%–5% potassium, 3% proteins, and approximately 1% important minerals, such as calcium, magnesium and phosphorous, which makes molasses an ideal substrate for biosurfactant production (Zhang et al., 2021). Table 3 summarizes various renewable substrates used for the production of biosurfactants.

**TABLE 3:** Renewable substrates used for biosurfactant production.

Biosurfactant	Microbial source	Substrates	Biosurfactant yield (g/L)	References
Lipopeptide	Halobacteriaceae *archaeon AS65.*	Banana peel	5.53	Chooklin et al. (2014)
ND^a^	*Candida sphaerica*	Ground-nut oil refinery residue	21	Luna et al. (2015)
Lipopeptide	*Bacillus subtilis BS6*	Soybean oil waste	1.2	Li et al. (2016)
ND^a^	*Aureobasidium thailandense*	Olive oil mill wastewater	0.14	Meneses et al. (2017)
Fatty acid glycoside	*Candida tropicalis*	Molasses, frying oil, and corn steep liquor	27	da Rocha Junior et al. (2018)
Surfactin	*Bacillus subtilis*	Clarified cashew apple juice	5.2	Nogueira Felix et al. (2019)
Surfactin	*Bacillus subtilis*	Trub (brewery waste)	0.21	Nazareth et al. (2020)
Glycolipid	*Mucor hiemalis*	Post-frying soybean oil	7.73	Ferreira et al. (2020)
Glycolipid	*Serratia nematodiphila*	Rice straw hydrolysates	4.9	Panjiar et al. (2020)
Surfactin	*Bacillus subtilis*	Pineapple peels	24.3	Jeevanandam et al. (2021)
ND^a^	*Pseudomonas frederiksbergensis*	Piggery wastewater	3.05	Guo and Gao (2021)
Rhamnolipid	*Pseudomonas aeruginosa*	Treated waste glycerol (by-product of biodiesel production)	11.32	Mahamad et al. (2025)
Surfactin lipopeptide	*Actinomycetes*	Mineral salt culture medium with 5% waste vegetable frying oil and 2.1% whey	1.64	Hesham et al. (2025)

^a^ ND-Not Determined.

### 4.2 Role of fermentation in biosurfactant synthesis

An essential strategy for enhancing biosurfactant production is selecting the most effective fermentation process. Biosurfactants are typically produced using either submerged fermentation (SmF) or solid-state fermentation (SSF). Selection of fermentation type is critical for optimizing yield, as each method creates distinct conditions for microbial activity and influences key factors, such as oxygen levels, nutrient availability, and water activity (Barbosa et al., 2021; Phulpoto et al., 2023).

The SmF process involves the growth of microbes, substrate utilization, and release of the desired biomolecules within the liquid media. At an industrial scale, biosurfactant production typically uses large aeration/agitation bioreactors. The yield and productivity of biosurfactants are influenced by several factors, including pH, temperature, incubation period, dissolved oxygen concentration, nutrients, and aeration rates. There are several advantages of SmF, such as precise instrumentation control over process parameters, including pH and temperature monitoring, dissolved oxygen regulation, and agitation (Barbosa et al., 2021; Phulpoto et al., 2023). Additionally, it simplifies downstream processing, such as biomass separation and product recovery. For example, Rastogi and Kumar, (2021) used SmF to produce 2.4 g/L of rhamnolipid from *Pseudomonas* sp. F5. Similarly, another study demonstrated that *Bacillus haynesii E1* produced 3.7 g/L of lipopeptide using this technology (Rastogi et al., 2021). However, there are some challenges associated with the use of SmF manufacturing biosurfactants, particularly the formation of excessive foam due to forced aeration and agitation. This foaming can result in significant losses of biomass, nutrients, and products, ultimately reducing the biosurfactant yield and extreme conditions rendering the entire operation unfeasible.

The SSF is an industrially attractive bioprocess that involves the growth of microbes on a solid surface that has the property of absorbing and retaining moisture without the requirement of soluble nutrients (Barbosa et al., 2021; Phulpoto et al., 2023). The low water volume in the SSF significantly influences the process economics due to streamlined downstream processing and utilization of inexpensive substrates as growth media, usually agricultural wastes. The SSF is advantageous over SmF because of its ability to overcome the foaming problem (Thomas et al., 2013; Pardhi et al., 2022; Phulpoto et al., 2023). Although requiring some optimization to overcome the challenges associated with heat and mass transmission in the reactors, SSF offers much more economical biosurfactant production. Several studies have reported a higher biosurfactant production in SSF than in SmF. For example, Sun et al., 2019 reported a four times higher iturin production in SSF than in SmF. Similarly, lipopeptide production was 3.5 times higher in SSF than SmF by strains *Bacillus velezensis* GA1 and *Bacillus subtilis* ATCC 21,332 (Valdés-Velasco et al., 2022). Therefore, the commercial applications of SSF are gaining more interest due to its low energy requirements, high output yields, and decreased risk of microbial contamination due to minimal wastewater production (Phulpoto et al., 2023; Cano y Postigo et al., 2021).

Furthermore, the fermentation processes of biosurfactants are also challenged by the generation of excessive foam due to surface activity and high agitation and aeration rates, which are usually required to effectively produce biosurfactant-producing microbes. Furthermore, high foaming can lead to product losses, reduce the productivity of the process, increase the cost of production, and reduce product quality (Gudiña and Teixeira, 2022; Leal et al., 2024). The use of physical means, such as mechanical disruptors, along with high energy consumption can also cause cell damage due to shear stress. However, chemical antifoaming agents can increase the costs of downstream processes and purification. Some chemical agents might impede the growth of microbes even at low volumes. Several other alternative techniques have been developed to avoid foaming, including exploring unconventional oxygen transfer bioreactors, such as membrane-aerated bioreactors or biofilm bioreactors. However, due to disadvantages, such as clogging of structural packing, limiting of oxygen caused by toxic product formation, complex purification and scale-up difficulty, these methods are not used widely (Gudiña and Teixeira, 2022; Leal et al., 2024; Hoffmann et al., 2021).

Another technique that has not been explored extensively is biosurfactant production under anaerobic conditions. A study showed that under oxygen-limiting conditions, cell viability and survival were improved for *B. subtilis* through surfactin formation (Arjes et al., 2020). However, Hoffmann et al. (2021) demonstrated that surfactin production was 20–33 times higher under aerobic rather than anaerobic conditions.

### 4.3 Advanced downstream processing for biosurfactant recovery and purification

A substantial portion of the production cost for biosurfactants is often attributed to downstream process unit operations, such as cell separation, biomass removal, and product recovery. The process should recover a stable, highly purified, and cost-effective product. Biosurfactant recovery and purification directly depend on the ionic charge, foaming capacity, solubility, molecular size, and if the biosurfactant is accumulated intracellularly or extracellularly (Najmi et al., 2018; Sundaram, et al., 2024). The traditional biosurfactant recovery methods, namely, non-membrane purification techniques, such as acid precipitation and solvent extraction, foam fractionation, and adsorption, face several challenges owing to the high costs of solvents, lengthy processing time, generation of large quantities of toxic waste product, and low extraction efficiency of solvents, leading to product losses and additional fees for purifying crude biosurfactants (Vicente et al., 2021; Jauregi and Kourmentza, 2019; Ambaye et al., 2021). Consequently, it is imperative to develop efficient, sustainable, and economical techniques.

Membrane-based separation techniques have shown promise for enhancing yields, improving product recycling, and reducing waste generation. Ultrafiltration, a widely used membrane-based filtration technique, has advantages over other physical separation methods by reducing shear stress, increasing purity and yield, and enabling room temperature operations-(Jauregi and Kourmentza, 2019; Vicente et al., 2021). This technique efficiently recovers, concentrates, and purifies biomolecules continuously according to their size and molecular weight.

Ultrafiltration leverages the CMC property of biosurfactants, enabling their aggregates to be retained by molecular weight cutoff (MWCO) membranes. The cutoff values are typically 3–5 times smaller than that of the target molecule and are a key parameter for efficient separation because a MWCO that is too low often results in lower recoveries. Additionally, selecting an appropriate membrane material is essential for minimizing disruption, ensuring efficient separation, reducing fouling, and maintaining process stability (Van der Bruggen, 2018; Vicente et al., 2021). Polyethersulfone (PES) membranes are particularly well-suited due to their high thermal and chemical resistance, excellent hydrolytic and mechanical stability, and low biosurfactant rejection, with their negatively charged surface further enhancing the flux (Hotza et al., 2020; Vicente et al., 2021). De Andrade et al. (2016) employed a two-step ultrafiltration process using a 100 kDa MWCO PES membrane to achieve a 68% surfactin yield.

Ceramic membranes are another promising material for ultrafiltration because of their high chemical resistance, broad pH tolerance, thorough cleanability, and extended lifespan, making them economical with minimal replacement requirements. Their application in biosurfactant purification remains largely unexplored (Lee et al., 2016; Vicente et al., 2021).

The mode of ultrafiltration operation is another important factor for optimizing purification operations. The primary modes of ultrafiltration include dead-end filtration (feed flow is perpendicular to membrane surface) and crossflow filtration (feed flow is parallel to membrane surface). Crossflow filtration is usually selected because it enables greater separation and recovery with minimal fouling (Yogarathinam et al., 2022; Wang et al., 2022). Rangarajan et al. (2014) used crossflow ultrafiltration for the recovery and purification of lipopeptides, specifically surfactin and iturin. Using polyethersulfone membranes with a MWCO of 50 kDa, they achieved a lipopeptide recovery rate of 90.5%.

Other factors, such as the rejection or retention rate and permeate flux, also affect the efficiency of ultrafiltration. The rejection or retention rate is the difference in the biosurfactant concentration between the retentate and permeate after ultrafiltration, whereas the permeate flux is the volume flowing through the membrane per unit of time. Furthermore, parameters, such as pH, transmembrane pressure, and the initial concentration of the solution may affect the ultrafiltration operations. Therefore, ultrafiltration is a highly efficient, sustainable, faster, and economical technique that can recover and purify ≤95% of biosurfactants (Vicente et al., 2021; Ambaye et al., 2021).

Nanofiltration is another emerging technology for biosurfactant purification, that uses membranes with pore sizes ranging from 1 to 10 nm for selective separation based on molecular weights and sizes. This technique operates at low pressure and temperature, ensuring efficient purification while preserving the biosurfactant functional properties (Vicente et al., 2021). Organic solvent nanofiltration has been successfully used to achieve 98% purity of mannosyl erythritol lipids (Nascimento et al., 2023). Although nanofiltration is cost- and energy-efficient, its industrial application is hindered by membrane fouling and high initial set-up costs.

Overall, the integration of low-cost agroindustrial waste substrates with membrane-based separation techniques is a cost-effective and scalable approach to biosurfactant production.

### 4.4 Genetic and metabolic engineering strategies

The synthesis of biosurfactants depends on the activity of specific enzymes and genes of microorganisms. Their production can vary according to the type of microbe and its particular genetic makeup. The adaptability of microorganisms and their ability to regulate biosurfactant production under several physicochemical conditions is due to their genetic diversity. Furthermore, the chemical nature of a biosurfactant is also affected by diverse genetic makeup of their hosts (Bazire and Dufour, 2014; Song et al., 2022; Chabhadiya et al., 2024). This relationship has led to researchers to analyze various classes of biosurfactant, and the genes associated with them for specific industrial and environmental roles. The dynamic process of biosurfactant production across different species and environments is highly influenced by environmental signals, microbial characteristics, and genetics.

To put this in perspective, the environmental cues and nutrient availability trigger the biosurfactant biosynthesis genes (Song et al., 2022; Lovaglio et al., 2023; Chabhadiya et al., 2024). Environmental changes are identified by the sensing mechanisms, which then enable the microbes to respond to various factors, such as oil or hydrophobic substrates. This in turn activates the regulatory genes that control the expression of biosynthetic genes to ensure that biosurfactant production is tailored to the environmental needs. These genes are organized within a biosurfactant operon, a cluster of genes that collectively regulate biosurfactant synthesis, secretion, and adaptation (Banat et al., 2010; Abbot et al., 2022; Chabhadiya et al., 2024). Within this operon, biosurfactant biosynthesis genes encode the enzymes and proteins necessary for the biosurfactant structure and activity. In addition, transport and secretion genes are essential for releasing biosurfactants into the environment, where they perform surface-active roles in applications, such as bioremediation and emulsification. The final step is environmental release, in which biosurfactants are discharged to reduce surface tension, form emulsions, and enhance microbial interaction with hydrophobic substrates (Danevčič et al., 2021; Song et al., 2022; Chabhadiya et al., 2024). This coordinated system ensures that microorganisms efficiently produce and utilize biosurfactants to adapt and survive under diverse environmental conditions.

Several genes have been identified across different microorganisms that have key roles in the synthesis of different classes of biosurfactants with intrinsic properties. Table 4 lists some of the common and uncommon genes related to the synthesis of different biosurfactants.

**TABLE 4:** Ubiquitous and rare genes associated with the biosynthetic pathways of various biosurfactants.

Gene name	Host microorganism	Genomic presence	Biological function	References
*rhlAB*	*Pseudomonas aeruginosa*	Ubiquitous	Encodes rhamnosyltransferase 1 (RhlA), catalyzing L-rhamnose transfer to HAA for mono-rhamnolipid biosynthesis.	Bazire and Dufour (2014)
*srfA*	*Bacillus subtilis*	Ubiquitous	Positively regulates surfactin biosynthesis by activating the srfA operon, which encodes surfactin-synthesizing enzymes.	Danevčič et al. (2021)
*ituD*	*Bacillus subtilis*	Ubiquitous	Encodes iturin A, mediating antimicrobial activity and surface tension reduction of the biosurfactant.	Song et al. (2022)
*LichenysinA*	*Bacillus licheniformis*	Ubiquitous	Encodes enzymes in the NRPS complex responsible for lichenysin A biosynthesis.	Xin et al. (2022)
*Mannosylerythritol lipid*	*Pseudozyma spp*	Ubiquitous	Involved in mannosylerythritol lipid (MEL) biosynthesis.	Fardami et al. (2022)
*CbsA*	*Bacillus clausii*	Rare	Encodes surface layer protein, enhancing cell surface hydrophobicity to facilitate biosurfactant production.	Jimoh et al. (2021)
*DofA*	*Pseudomonas fluorescens*	Rare	Involved in dirhamnolipid biosynthesis by catalyzing the addition of a second L-rhamnose unit to mono-rhamnolipid.	Kusuma et al. (2021)
*emulsanB*	*Acinetobacter cacoaceticus*	Rare	Responsible for the biosynthesis of the biosurfactant emulsan.	Dias and Nitschke (2023)
*KrmA*	*Bacillus thuringiensis*	Rare	Encodes enzymes responsible for kurthiosurfactin biosynthesis, a lipopeptide biosurfactant with surface-active and antimicrobial properties.	Song et al. (2022)
*TspR*	*Pseudomonas fluorescens*	Rare	Involved in tensin biosynthesis, contributing to its surfactant and emulsifying properties.	Jimoh et al. (2021)
*LtpR*	*Lysinibacillus fusiformis*	Rare	Plays a key role in tensin biosynthesis, facilitating its surfactant and emulsification functions.	Md Badrul Hisham et al. (2019)

Metabolic engineering and synthetic biology are powerful tools for modifying biosurfactant-producing genes to create tailored biosurfactants with properties specific to their application, leading to optimized operations (Danevčič et al., 2021; Lovaglio et al., 2023; Chabhadiya et al., 2024). Genetic and metabolic engineering can help overcome the challenges associated with industrial production by optimizing carbon utilization by modifying biosynthetic pathways, streamlining metabolic fluxes, minimizing byproduct formations, and decoupling biosurfactant synthesis from native cellular regulation (Toribio et al., 2010; Bahia et al., 2018; Lovaglio et al., 2023). By leveraging heterologous hosts, the limitations associated with pathogenic strains, such as *Pseudomonas aeruginosa*, *can* be circumvented to facilitate a safer and scalable operation. The following strategies can be applied to enhance biosurfactant yield and streamline large-scale production.

- Regulation and Heterologous Expression of Biosurfactant Genes: The intricate regulatory network governing biosurfactant synthesis poses a significant challenge for industrial scale production. Quorum sensing is a key regulatory mechanism that tightly controls biosurfactant biosynthesis and other cellular processes, often leading to suboptimal yields under industrial conditions (Toribio et al., 2010; Lovaglio et al., 2023). Various strategies have been explored to bypass these regulatory constraints, with heterologous expression emerging as the most promising approach. This strategy involves the transfer of biosurfactant-producing genes into non-pathogenic, industrially robust hosts, such as *E. coli* and *Pseudomonas putida*, enabling safer and more efficient production (Bahia et al., 2018; Lovaglio et al., 2023). A previous study introduced the rhlAB operon, which is responsible for rhamnolipid biosynthesis, into *P. aeruginosa* PAO1-rhlA and *Escherichia coli* BL21 under the control of a T7 promoter. The engineered *E. coli* strain demonstrated a rhamnolipid production yield of 167.5 mg L^−1^, highlighting the potential of heterologous systems for biosurfactant synthesis (Wang et al., 2007). Genomic integration of a glycosyltransferase gene from *Pseudomonas* into *Bacillus subtilis* enhanced glycolipid biosynthesis and hydrocarbon degradation. After 7 days, the engineered *Bacillus–Pseudomonas* co-culture increased crude oil degradation from 32.61% to 54.35% (individual strains) to 63.05%, demonstrating synergistic improvement in bioremediation efficiency (Wu et al., 2023). Furthermore Zhao et al. (2021), demonstrated that the overexpression of key biosynthetic genes (*rmlBDAC*, *rhlABRI*, and *rhlC*) in *P. aeruginosa* SG significantly enhanced anaerobic rhamnolipid production. The engineered strain *P. aeruginosa* SGhm achieved a yield of 1.34 g/L which was about 4.5-fold higher than the wild type (0.24 g/L). This was further increased to 1.54 g/L after medium optimization using response surface methodology. The strain efficiently emulsified oil under anaerobic conditions, producing 89.4% of oil droplets with diameters between 0 and 5 μm.
- Optimizing Substrate Utilization for Cost-Effective Production: The cost of biosurfactant production is mostly influenced by substrate expenses, making the use of renewable, waste-derived feedstocks a viable and sustainable alternative to traditional carbon sources. Agro-industrial residues and dairy effluents offer promising low-cost substrates; however, their complex composition limits their direct utilization by native biosurfactant-producing microbes (Kawaguchi et al., 2016; Vu et al., 2020; Lovaglio et al., 2023). To overcome this, metabolic engineering enables the introduction of heterologous transporters and enzyme genes into host strains, expanding their substrate range. For example, Meijnen et al. (2009) enhanced *P putida* S12 by integrating *xylA* (xylose isomerase) and *xylB* (xylulose kinase) genes from *E. coli*, enabling efficient metabolism of xylose and L-arabinose as carbon sources for biosurfactant synthesis.
- Cellular physiology optimization: Biosurfactant production can be enhanced by optimizing overall cellular physiology. Various techniques are used, such as random mutagenesis (exposure of a strain to UV or chemicals resulting in mutant strains), adaptive laboratory evolution (evolution under selective pressures), and directed genetic mutations (precise and targeted alterations in the genome to modify traits) (Dobler et al., 2016; He et al., 2017; Lovaglio et al., 2023). Overexpression of the estA gene in *P. aeruginosa* increases the production of rhamnolipid by approximately four times (Dobler et al., 2017).

Therefore, application of metagenomics, bioinformatics, gene editing, DNA sequencing, proteomics, and metabolomics can optimize the production and yield of biosurfactants at an industrial scale by enhancing existing strains or identifying novel biosynthetic pathways.

### 4.5 Nanoparticle-assisted formulations for enhanced biosurfactant applications

In recent years, nanoparticle–biosurfactant formulations have gained considerable attention over their traditional counterparts due to their superior stability, reusability, and ecofriendly profile. These hybrid formulations enhance key processes in bioremediation and EOR by improving oil dispersion, facilitating microbial degradation, and increasing hydrocarbon removal efficiency (El-Sheshtawy et al., 2017; Elumalai et al., 2024). In bioremediation, nanoparticle–biosurfactant systems demonstrate high stability and efficiency, with studies reporting ≤84% hydrocarbon removal. For example, Elumalai et al. (2024) observed that a formulation comprising a biosurfactant, a mixed microbial consortium (*Bacillus subtilis* R6, and *P. dendritiformis* S2), and iron-oxide nanoparticles achieved 99% efficiency under optimal conditions. Similarly, El-Sheshtawy et al. (2017) reported 90% biodegradation using the biosurfactant–nanoparticle consortia. Notably, these formulations can be recycled and reused, as seen in a study in which Fe–Cu nanoparticles with rhamnolipids retained 59% efficiency even after three bioremediation cycles (Vu and Mulligan, 2022). Furthermore, nanoparticles enhance heavy-metal sequestration, as biosurfactant-capped nanoparticles produce ultra-fine stable particles that effectively chelate heavy metals. This dual functionality not only improves metal uptake but also prevents nanoparticle aggregation, making them viable for wastewater treatment without secondary pollution. For example, rhamnolipid-coated iron-oxide nanoparticles efficiently absorb uranium (VI) from contaminated soil (Sharma et al., 2020).

Beyond bioremediation, nanoparticle–biosurfactant systems have revolutionized EOR by leveraging their synergistic effects in IFT reduction and rock wettability alteration. In one study, a combination of emulsan, a biosurfactant, and silica nanofluids reduced the oil–water IFT by 90%, nearly doubling the oil recovery in a glass micromodel (Amani, 2017). Similarly, Janaghi et al. (2022) demonstrated that using rhamnolipids in combination with N-graphene at the CMC resulted in 24.2% original oil in place recovery and a 90% reduction in IFT, highlighting the efficiency of nanoparticle–biosurfactant hybrids in oil displacement. However, these innovative formulations must meet stringent performance criteria, particularly stability, efficacy, and reproducibility across diverse temperatures, pH levels, and other challenging environmental conditions typically encountered in oil refinery operations (El-Sheshtawy et al., 2017; Aslam et al., 2024; Elumalai et al., 2024).

Integration of nanoparticles with biosurfactants offers a sustainable and highly efficient alternative to conventional methods in bioremediation and EOR. As research advances, optimizing these hybrid systems for cost-effectiveness and field-scale implementation will be crucial for their widespread adoption in the environmental and petroleum engineering sectors.

### 4.6 Integration of artificial intelligence in biosurfactant research and production

For many years, AI models have been used in the petroleum industry for various applications, such as improving operational efficiencies and utilizing resource. AI-driven models, such as artificial neural networks (ANNs), have used multidimensional data from traditional techniques and have essentially transformed them by providing predictive insights into various challenges and processes. AI is very effective in analyzing the rock properties in the reservoir, including porosity, permeability, and lithology, by integrating data from logs, seismic surveys, and laboratory experiments to develop accurate reservoir models for better yields (Mohaghegh, 2011; Feng, 2020). In EOR, AI has optimized traditional techniques, such as gas injection and water and chemical flooding techniques by predicting the interaction of the EOR agents and the reservoir fluid and rocks (Elkatatny et al., 2018; Nnadili et al., 2024). Furthermore, with the help of sensor networks and monitoring systems, it is now easy to predict, detect, and prevent early signs of corrosion, leaks, or mechanical strains, which can lead to operational anomalies. Additionally, AI plays a pivotal role in the management of wax deposition by predicting the precipitation conditions on the basis of the fluid properties, temperature, and pressure while optimizing the use of inhibitors, heating systems, or pigging schedules to mitigate disruptions. Thus, it is observed that AI is having an integral role in the petroleum industry (Alamri, 2022).

Similarly, AI is providing innovative solutions for optimizing and scaling up the production of biosurfactants. As biosurfactants are microbial-derived amphiphilic molecules, complete control is essential over all the production parameters to achieve higher yields, low production cost, and desired functional properties (Pal et al., 2009; da Silva et al., 2024). AI models facilitate these by analyzing vast datasets for different lab-scale experiments and upstream and downstream processes to provide effective predictive insights and process optimization strategies. With the help of AI, the increase in the yield of biosurfactants can be achieved through various pathways, including media optimization, strain selection, engineering, fermentation parameter optimization, substrate selection, prediction of scale-up conditions, and real-time monitoring of biosurfactant properties (Saraç et al., 2022; da Silva et al., 2024).

ANNs, a nonlinear multivariate modeling tool that mimics the data-processing mechanisms of the human brain, are gaining popularity over traditional statistical models (Ekpenyong et al., 2020). ANNs are based on interconnected layers of neurons in which an input layer receives information, a hidden layer analyzes relationships and interactions among variables, and an output layer provides predictions or suggested values. (Ekpenyong et al., 2020). For example, Sivapathasekaran et al. (2010) used ANN and genetic algorithm modeling to achieve a 70% enhancement in the biosurfactant production by *Bacillus circulans* MTCC 8281. Through this single-factor-at-a-time optimization strategy, the study identified the critical medium components of glucose, urea, SrCl_2_, and MgSO_4_. Similarly, in another study, ANN methods predicted that the maximum glycolipid concentration was achieved under optimal fermentation conditions of 32 °C, pH 7.6, 130 rpm agitation, and 66 h (Ekpenyong et al., 2020 *).* ANN-GA optimization has also proven to be effective in enhancing tertiary oil recovery by ≤45% by recommending the use of a *Bacillus licheniformis*-produced biopolymer as a flooding agent in combination with a lipopeptide (1,000 mg/L), Ca^2+^ (80 mg/L), and a pH of 7.2 (Dhanarajan et al., 2017).

Deep learning (DL), which is a subset of artificial intelligence and an extension of artificial neural networks, employs multiple hidden layers to model complex, non-linear relationships within large datasets. Unlike conventional ANN models, DL can automatically extract high-level features from raw data, enabling improved accuracy and adaptive learning for process optimization in dynamic industrial environments (Haohao et al., 2024). The introduction of DL into oil and gas development can be advantageous, offering novel approaches to solving complex problems. For instance, during the exploration stages of petroleum, DL can assist in the in-depth analysis and interpretation of seismic data to better understand the state of underground reservoirs, thereby improving the accuracy and efficiency of reservoir production. Furthermore, during the production stage, deep analysis of production data combined with real-time monitoring of key process parameters can help optimize well performance (Haohao et al., 2024).

Reinforcement Learning (RL) is another advanced machine learning algorithm that learns optimal decision-making strategies through iterative interactions with its environment. The models interpret historical and real-time data to provide insights that identify optimal process parameters to increase efficiency and yield. RL offers continuous adaptability to changes in feedstock quality and environmental conditions, enabling the system to maintain optimal performance under variable operational scenarios (Butean et al., 2025).

Digital twins (DT) are virtual replicas of physical systems that integrate real-time operational data with predictive computational models. Digital twin modeling can be incorporated into all aspects of the hydrocarbon industry, beginning with site surveys and extending through exploration, evaluation, production, performance monitoring, and equipment replacement. DT can optimize exploration processes by improving productivity, reducing health, safety, and environmental (HSE) risks, and minimizing capital and operational costs, thereby increasing overall revenue. Additionally, DT can enhance digital reservoir models to simulate flow, transport, and geomechanical behavior in subsurface porous media, as well as assess groundwater contamination, thus improving process efficiency and environmental monitoring. The implementation of DT in real-time operations, such as drilling, can further enhance performance by automatically detecting operational difficulties and addressing potential hazards before their occurrence. Moreover, DT strategies improve data optimization and decision-making by integrating multi-source datasets, facilitating predictive analytics, and enabling continuous feedback loops between virtual models and physical systems (Sircar et al., 2023).

Thus, in conclusion, the integration of AI, ANN, Deep Learning (DL), Reinforcement Learning (RL) and Digital Twins in biosurfactant production helps to enhance its yield by optimizing the process parameters, leading to more efficient, less time-consuming, cost-effective, and sustainable methods for scale-up.

## 5 Future prospects and conclusion

Given their properties (e.g., surface tension reduction, emulsification, demulsification, stability across various temperature and pH ranges, biodegradability, and biocompatibility), biosurfactants are valuable tools in the petroleum sector (Esmaeili et al., 2021; Al-Sakkaf and Onaizi, 2023). Their potential effects are expected to grow exponentially, as indicated by the increasing number of patents on their applications to enhance operations, environmental sustainability, or production of alternate fuels for energy. The global biosurfactant market was valued at USD 4.41 billion in 2023 and is projected to grow exponentially to a staggering USD 6.71 billion by 2032 exhibiting a CAGR of 5.4% (Biosurfactants market size, share, trends, 2025). Sultana et al., 2024, reported that rhamnolipid biosurfactant produced from an oil-tolerant strain, *Bacillus velezensis S2* was isolated from an oil contaminated site which led to >50% degradation of convoluted crude oil within 28 days in comparison to a control. Similarly, an field study carried out in the Yingdong Oilfield, Huatugou Oilfield and Yuejin Oilfield revealed cumulative increase of 4,573.82 m^2^ when biosurfactant produced by *Bacillus velezensis* B6 was used as a oil displacement agent (Chen et al., 2025). REWOFERM® SL ONE, developed by Evonik Industries, is a novel sophorolipid-based biosurfactant produced through the fermentation of sugar beet–derived sugars and rapeseed oil using the yeast *Candida bombicola*. It is a fully bio-based, biodegradable surfactant that can be applied as a membrane cleaner, hard surface cleaner, and metal cleaner in various industrial formulations (Evonik).

However, there are major limitations regarding their commercialization (de Almeida et al., 2016; Nikolova and Gutierrez, 2021). These limitations include the high cost of raw materials for biosurfactant production and downstream processing, low yields, and, in some cases, extreme foaming. In addition, biosurfactant-producing microorganisms, such as *P. aeruginosa*, are pathogenic and can produce virulence factors. Moreover, some biosurfactants reportedly have biocidal effects on microbes essential for hydrocarbon degradation. Biosurfactants that are effective in the laboratory-scale bioremediation of petroleum contaminants are ineffective in pilot-scale oil-spill management (Uttlová et al., 2016; Dhanya, 2021).

Therefore, to achieve effective commercialization of biosurfactants, strategies must be aimed at increasing productivity and reducing production costs. Some techniques that can be incorporated include using waste as a raw material for biosurfactant production, optimizing media formulations, aeration systems, fermentation technologies, and downstream processing for increased production, as well as employing genetic engineering technologies, such as molecular cloning and CRISPR-Cas9, to develop hyper–biosurfactant-producing microbes (Wittgens et al., 2016; Nikolova and Gutierrez, 2021). Governments can further accelerate the commercialization of biosurfactants by offering financial incentives, such as research grants and subsidies, to support production scale-up and innovation. Implementing favorable tax policies or exemptions for businesses investing in the development of sustainable biosurfactant technologies can significantly improve their market competitiveness. Governments can also streamline regulatory-approval processes, establish clear public–private partnerships, and adopt biosurfactants in public procurement policies. Collectively, these measures would foster industry collaboration, drive market adoption, and stimulate broader innovation in sustainable biosurfactant applications.

In conclusion, continued research, industrial collaboration, and technological advancements will be pivotal for harnessing the full potential and subsequent commercialization of biosurfactants in the petroleum sector.
